# Impact of Pulmonary Hypertension on Posttransplant Survival of Patients With Pulmonary Fibrosis at High Altitude: A Prospective Cohort Study

**DOI:** 10.1155/carj/1861990

**Published:** 2025-02-24

**Authors:** Fabio Varón-Vega, Luis J. Telléz, Eduardo Tuta-Quintero, Adriana Rincón, Diana Casas, Camilo Rodriguez, David Mendoza, Luis Fernando Giraldo-Cadavid

**Affiliations:** ^1^Critical Care and Lung Transplantation Service, Fundación Neumológica Colombiana, Fundación Cardio Infantil–Instituto de Cardiología, Bogotá, Colombia; ^2^Critical Care Service, Fundación Neumológica Colombiana, Fundación Cardio Infantil–Instituto de Cardiología, Bogotá, Colombia; ^3^Thoracic Surgery Service, Fundación Cardio Infantil–Instituto de Cardiología, Bogotá, Colombia; ^4^School of Medicine, Universidad de La Sabana, Chía, Colombia; ^5^Interventional Pulmonology Service, Fundación Neumológica Colombiana, Bogotá, Colombia

**Keywords:** lung transplant, pulmonary fibrosis, pulmonary hypertension, survival

## Abstract

**Background:** Pulmonary hypertension (PH) in patients undergoing lung transplantation (LT) for pulmonary fibrosis can impair lung function, reduce physical activity, and decrease survival. However, data on outcomes at 1 and 5 years of follow-up remain limited.

**Methods:** In this prospective cohort study, pulmonary function, the 6-min walk test (6MWT), and the St. George's Respiratory Questionnaire (SGRQ) were assessed pretransplant, at hospital discharge, and at 3, 6, and 12 months posttransplant. Additionally, minimal clinically important differences (MCIDs) between patients with and without PH were evaluated. Survival rates were calculated using the Kaplan–Meier method and analyzed using the log-rank test.

**Results:** The study included 39 patients undergoing LT for pulmonary fibrosis. Of these, 82% (32/39) had PH, with a median age of 52.6 years (SD: 10.2). In both the PH and non-PH groups, lung function, 6MWD, and SGRQ total scores showed progressive improvement from pre-LT to 1 year posttransplant. Patients without PH demonstrated MCID in 6MWT and SGRQ total scores from pre-LT through the 6- and 12-month follow-up. The overall 1-year survival rate was 84.6%, with an average survival of 10.51 months (95% CI: 9.29–11.73). The 5-year overall survival rate was 61.5%, with an average survival of 44.89 months (95% CI: 37.62–52.16). No statistically significant differences in survival were found based on sex (*p*=0.322 and 0.206), mean pulmonary artery pressure (mPAP) (*p*=0.232 and 0.486), age (*p*=0.375 and 0.959), or body mass index (BMI) (*p*=0.884 and 0.594) at 1 and 5 years.

**Conclusion:** Survival at 1 and 5 years was lower in patients with PH. However, no significant differences in survival were observed based on sex, mPAP, age, or BMI. Statistically significant improvements in FVC, FEV1, 6MWT, and SGRQ total scores were observed both before and after LT, continuing through 1 year of follow-up. The 6MWT and SGRQ showed MCID both prior to surgery and during follow-up at 6 and 12 months, in both PH and non-PH patients.

## 1. Introduction

Lung transplantation (LT) is a definitive surgical intervention for treating advanced or end-stage lung diseases [1]. The evaluation and selection process for transplantation must minimize perioperative risks and positively impact posttransplant survival [2, 3]. The shortage of lung donors and the high rate of complications during the transplantation process necessitate the identification of suitable candidates and the implementation of therapeutic strategies before, during, and after the transplant [3–5]. These strategies include immunosuppression, modern surgical techniques, the use of extracorporeal circulation, and the management of comorbidities, all aimed at achieving satisfactory clinical outcomes [4, 5]. Pulmonary hypertension (PH) is a common comorbidity in patients with end-stage fibrotic lung diseases undergoing LT, which can lead to decreased survival due to associated complications during surgery and in the posttransplant period [6–8].

Pulmonary fibrosis (PF) is a disease characterized by progressive scarring of the lung interstitium, which impairs lung function and quality of life [9]. In patients with advanced PF, LT is a therapeutic option that has been shown to improve survival [4, 5, 8, 9]. Briceño et al. [10] compared posttransplant survival in 35 patients diagnosed with PF, both with and without PH, and found no significant differences (*p*=0.74) in survival at 90 days and 12 months after LT. However, PH can serve as a predictor of disease severity, increased mortality in patients on the transplant list, and decreased survival in the posttransplant period [11–13].

Patients undergoing LT frequently present with PH and are at risk for short- and long-term complications [8, 14, 15]. Stącel et al. [16] reported a 60.1% (86/143) prevalence of PH in patients scheduled for LT. Wrobel et al. [17] described the clinical outcomes post-LT in 46 patients with chronic obstructive pulmonary disease who had documented PH prior to surgery. They observed a longer duration of mechanical ventilation (*p*=0.024), a poorer arterial oxygen pressure/fraction of inspired oxygen ratio at 24 h (*p*=0.027), and a longer stay in the intensive care unit (*p*=0.055). Furthermore, the hemodynamic repercussions of PH, along with right ventricular hypertrophy and relative underfilling of the left ventricle, induce significant changes in the early postoperative period [7, 14]. This hemodynamic adaptation substantially increases the risk of pulmonary edema and primary graft dysfunction [14, 15, 18].

Several studies have investigated high-altitude tolerance in transplanted patients [19, 20]. Gieszer et al. [21] conducted periodic evaluations during high-altitude walks and described a general and cardiopulmonary status in LT patients that was similar to or even superior to a control group of healthy individuals. However, there is a lack of evidence addressing the survival and clinical outcomes of transplantation in patients with PH at altitudes above 1500 m above sea level [22–25]. Consequently, our study aims to describe survival, quality of life, and functional test outcomes in patients with PF and PH undergoing LT at high altitude.

## 2. Methods

A prospective cohort study was conducted, including patients who underwent LT with a prior diagnosis of PH between 2014 and 2023 at the Fundación Cardioinfantil-Fundación Neumológica Colombiana, located in Bogotá, Colombia, at an elevation of 2640 m above sea level. The primary objective of the research was to evaluate survival at 1 and 5 years postprocedure. Additionally, the impact of PH before and after transplantation was analyzed in terms of pulmonary function tests, the 6-min walk test (6MWT), and quality of life.

### 2.1. Patient Selection

Patients over 18 years old who received an LT for PF, including allergic alveolitis, Sjögren's syndrome, rheumatoid lung, sarcoidosis, hypersensitivity pneumonitis, noninfectious nonspecific interstitial pneumonia (NSIP), idiopathic pulmonary fibrosis (IPF), acute respiratory distress syndrome (ARDS), and lymphangioleiomyomatosis, were included. Exclusion criteria included patients without survival data, those without conclusive echocardiographic findings of PH, and those in whom PF was ruled out in the explant.

### 2.2. Variables and Data Collection

The variables considered in the study included age, sex, body mass index (BMI), and the presence of PH, defined as a mean pulmonary arterial pressure (mPAP) > 20 mmHg, measured via right heart catheterization before the procedure [7]. Various LT-related variables were collected, including the type of transplant (single or double lung), ischemia time of the transplanted lungs, surgery duration, use of extracorporeal support, administration of blood products, use of inotropes, duration of mechanical ventilation, and length of stay in the intensive care unit, as well as intraoperative, immediate, and late complications. Functional test data were also collected, including forced vital capacity (FVC), forced expiratory volume in the first second (FEV1), and the distance covered in the 6MWT. To assess quality of life, the St. George's Respiratory Questionnaire (SGRQ) was used.

### 2.3. Peritransplant Rehabilitation

The pulmonary rehabilitation process was based on a comprehensive approach aimed at improving the patient's physical condition, optimizing respiratory capacity, and enhancing quality of life [26–28]. The training was tailored to each patient's individual capabilities and included endurance exercises such as walking or stationary cycling, performed either continuously or in intervals, along with strength and flexibility training. These exercises aimed to improve aerobic capacity, exercise tolerance, musculoskeletal efficiency, and reduce symptoms. During pretransplant rehabilitation, patients trained between 30 min and 1 hour daily, with a minimum of two sessions per week for at least 1 year before the transplant. In the posttransplant period, patients were rehabilitated for at least 6 months. In addition to physical rehabilitation, other key interventions in this integrated approach included psychological and nutritional support, both essential for optimizing transplant outcomes and improving recovery and quality of life [26–28].

To reduce reporting bias, at least two team members reviewed the information. In case of inconsistency, a third team member reviewed the data and made the final decision. Subjects were selected through convenience sampling from the list of patients seen during the study period. A missing data imputation analysis was performed for variables with less than 10% loss, applying weighted mean imputation for quantitative variables and logistic regression for qualitative variables [29]. A comparison between the nonimputed and imputed results confirmed that there were no significant differences that altered the original data [29]. Variables with more than 10% data loss were excluded from the analysis [29].

### 2.4. Statistical Analysis

Quantitative variables were summarized using measures of central tendency and dispersion, with means and standard deviations (SDs) for normal distributions, and medians and interquartile ranges (IQR) for nonnormal distributions. The Shapiro–Wilk test was used to assess normality. An analysis of clinical outcomes (FEV1, FVC, 6MWT, and SGRQ) was performed before the transplant, at hospital discharge, and at 3, 6, and 12 months posttransplant, using ANOVA or the Friedman test for repeated measures. A post hoc analysis was conducted using the Wilcoxon test or the Nemenyi test [29]. A bivariate analysis was performed using Student's *t*-test or the Mann–Whitney *U* test to evaluate the association between the presence or absence of PH and the clinical variables FEV1, FVC, 6MWT, and SGRQ total score.

The minimal clinically important difference (MCID) for functional variables was established before transplantation, at hospital discharge, and during clinical follow-up for patients with and without PH: an increase in FEV1 greater than 100 mL, an increase in FVC greater than 200 mL or 10%, a change in the distance covered in the 6MWT greater than 30 m, and an improvement in quality of life according to the SGRQ greater than 4 units [30–33]. One- and 5-year survival was assessed using the Kaplan–Meier method, with the log-rank test to evaluate statistical differences in survival curves according to independent variables [29]. All associations were considered statistically significant with a *p* value < 0.05 (two-tailed). The statistical analyses were performed using Microsoft Excel 2017 (Microsoft Corporation, Redmond, WA, USA) and Stata Version 17.0 (StataCorp LLC, College Station, Texas, USA).

### 2.5. Ethics Approval and Consent to Participate

The studies involving human participants were reviewed and approved by the Ethics Committee of the Fundación Cardioinfantil-Instituto de Cardiología. Prior to participation, all participants provided written informed consent, and the confidentiality of their data was strictly maintained throughout the study.

## 3. Results

### 3.1. Sociodemographic Analysis

The study included 39 patients undergoing LT with a diagnosis of PF. Of these, 82% (32/39) had PH, with a median age of 52.6 years (SD: 10.2). In the PH group, 50% (16/32) were men, compared to 57.1% (4/7) in the control group. IPF was the most common diagnosis in the PH group, accounting for 34.4% (11/32), compared to 42.8% (3/7) in the non-PH group. The characteristics of the population are described in Table 1.

### 3.2. Transplant Characteristics and Complications

In the PH group, 84% (32/39) underwent bilateral lung transplants, compared to 100% (7/7) in the control group (Table 2). The surgical duration was 340 min (IQR: 305–385) in the PH group versus 354 min (IQR: 330–420) in the control group. All patients in the non-PH group (100%, 7/7) experienced no intraoperative complications, compared to 67% (21/32) in the PH group. Furthermore, 86% (6/7) of the non-PH group had no immediate complications, compared to 67% (21/32) in the PH group. Regarding late complications, aspergillosis was the most frequent infection in the PH group, occurring in 15% (5/32) of cases.

### 3.3. Pulmonary Function Tests, 6MWT, and Quality of Life

**Figure 1:**
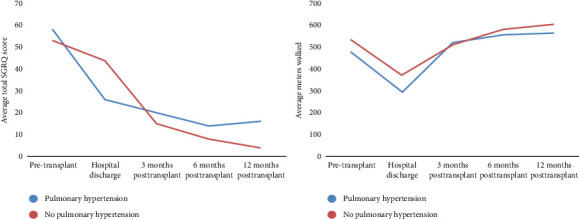
The St. George's Respiratory Questionnaire and 6-min walk test during the pre- and posttransplant period.

In the PH group, there was a progressive increase in FVC measurements from before LT (2.09 L; IQR: 1.5–2.7), at hospital discharge (2.18 L; IQR: 1.9–2.8), at 3 months (2.7 L; IQR: 2.1–3.3), at 6 months (2.98 L; IQR: 2.5–3.5), and at 12 months (2.92 L; IQR: 2.3–3.4) (Table 3). Quality of life, as measured by the SGRQ total score, showed a progressive improvement with scores decreasing from before LT (58.06 points; IQR: 44.5–68.5) to 12 months (16.48 points; IQR: 6.5–31.6). The changes in the SGRQ total score and 6MWT during the pre- and posttransplant periods are shown in Figure 1. Post hoc analysis of functional variables and quality of life is presented in Supporting Table 1.

### 3.4. MCID in Patients With and Without PH

Patients without PH had greater walking distances before surgery (MCID: 55 m), at hospital discharge (MCID: 76.5 m), at 6 months (MCID: 24.5 m), and at 12 months (MCID: 39.5 m). Regarding the total SGRQ total score, patients without PH showed more significant improvements, with lower scores before surgery (MCID: −4.8 points) and at 3 months (MCID: −4.6 points), 6 months (MCID: −5.5 points), and 12 months (MCID: −12.2 points). The MCID of pulmonary function is described in Supporting Table 2.

### 3.5. Survival Analysis

**Figure 2:**
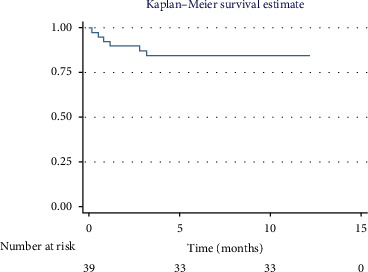
Overall survival in the first year.

**Figure 3:**
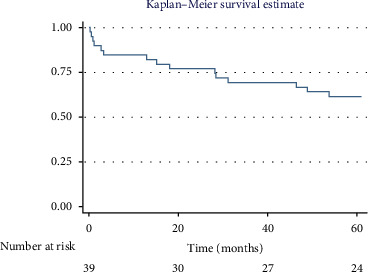
Overall survival in the fifth year.

The overall 1-year survival rate was 84.6%, with an average survival of 10.51 months (95% CI: 9.29–11.73) (Figure 2). The 5-year overall survival rate was 61.5%, with an average survival of 44.89 months (95% CI: 37.62–52.16) (Figure 3).

#### 3.5.1. One-Year Survival Analysis

**Figure 4:**
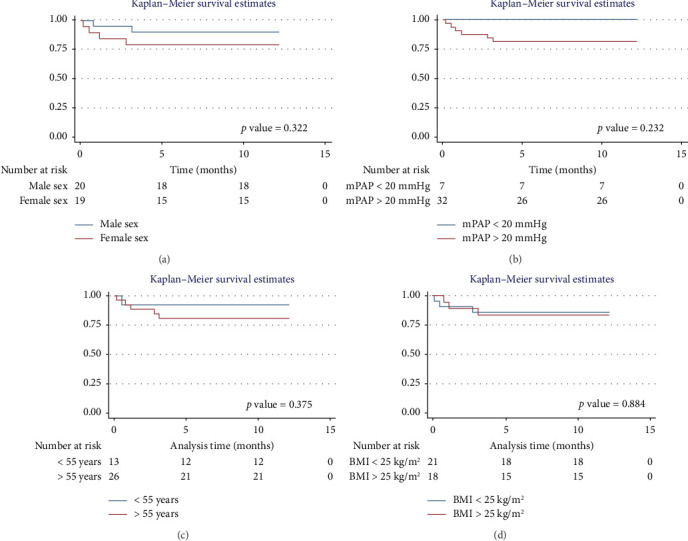
Survival by sex, mean pulmonary arterial pressure, age, and body mass index in the 1 year of follow-up. Notes: (a) survival stratified by sex. (b) Survival stratified by mean pulmonary arterial pressure. (c) Survival stratified by age. (d) Survival stratified by body mass index.

For men, the 1-year survival rate was 90%, with an average survival of 11.1 months (95% CI: 9.79–12.49) (Figure 4(a)). For women, the survival rate was 79%, with an average survival of 9.8 months (95% CI: 7.82–11.87) (*p* value: 0.322). In the PH group, 1-year survival was 81%, with an average survival of 10.15 months (95% CI: 8.69–11.62) (Figure 4(b)). Non-PH patients had a 100% survival rate, with an average survival of 12 months (*p* value: 0.232). Among patients older than 55 years, the 1-year survival rate was 80%, with an average survival of 10.1 months (95% CI: 8.52–11.74) (Figure 4(c)). For patients younger than 55 years, the survival rate was 92%, with an average survival of 11.2 months (95% CI: 9.58–12.95) (*p* value: 0.375). Regarding BMI, patients with a BMI > 25 kg/m^2^ had a 1-year survival rate of 83%, with an average survival of 10.4 months (95% CI: 8.61–12.23) (Figure 4(d)), while those with a BMI < 25 kg/m^2^ had a survival rate of 86%, with an average survival of 10.5 months (95% CI: 8.93–12.25) (*p* value: 0.884).

#### 3.5.2. Five-Year Survival Analysis

**Figure 5:**
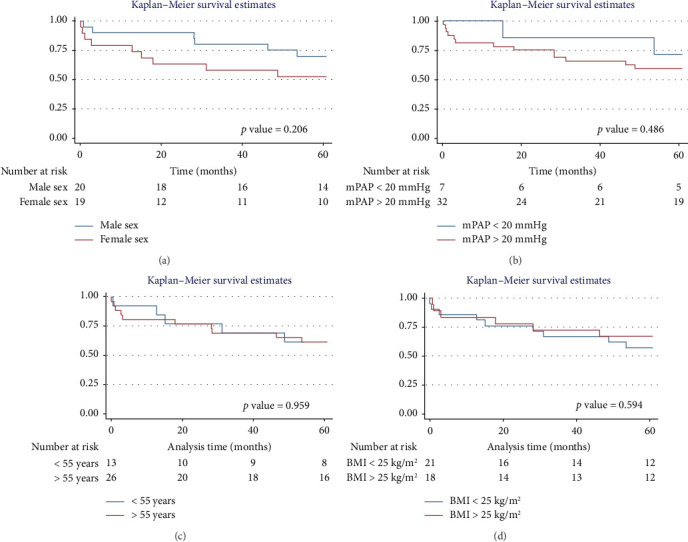
Survival by sex, mean pulmonary arterial pressure, age, and body mass index in the 5 years of follow-up. Notes: (a) survival stratified by sex. (b) Survival stratified by mean pulmonary arterial pressure. (c) Survival stratified by age. (d) Survival stratified by body mass index.

For men, the 5-year survival rate was 70%, with an average survival of 50.6 months (95% CI: 42.28–58.91) (Figure 5(a)). For women, the 5-year survival rate was 52.6%, with an average survival of 38.89 months (95% CI: 27.42–50.35) (*p* value: 0.206). In the PH group, the 5-year survival rate was 59.4%, with an average survival of 43.07 months (95% CI: 34.72–51.41) (Figure 5(b)). Non-PH patients had a 71.4% 5-year survival rate, with an average survival of 53.27 months (95% CI: 41.59–64.95) (*p* value: 0.486). For patients > 55 years, the 5-year survival rate was 61%, with an average survival of 44.4 months (95% CI: 35.28–53.62) (Figure 5(c)). For patients < 55 years, the 5-year survival rate was also 61%, with an average survival of 45.77 months (95% CI: 33.99–57.55) (*p* value: 0.959). In terms of BMI, patients with a BMI > 25 kg/m^2^ had a 5-year survival rate of 67%, with an average survival of 45.98 months (95% CI: 35.29–56.66) (Figure 5(d)), while those with a BMI < 25 kg/m^2^ had a survival rate of 57%, with an average survival of 43.96 months (95% CI: 34.07–53.86) (*p* value: 0.594).

## 4. Discussion

This study describes the survival and the impact of functional clinical variables in patients with PF who underwent pretransplant cardiac catheterization with mPAP > 20 mmHg. A 1-year survival rate of 84.6% was observed, with an average survival of 10.5 months, and a 5-year survival rate of 61.5%, with an average survival of 44.9 months. Survival at 1 and 5 years was lower in patients with PH. No significant differences in survival were found based on sex, age, or BMI. On the other hand, statistically significant improvements were observed in FVC, FEV1, 6MWT, and SGRQ measurements both before and after LT, which continued through 1 year of clinical follow-up. The 6MWT and SGRQ total score showed MCID before the surgical procedure and during clinical follow-up at 6 and 12 months among patients with and without PH. The frequency of intraoperative and immediate complications was higher in the PH group. Our study provides an overview of the impact of PH in patients undergoing LT at high altitude.

PH is a complex condition that can complicate LT and negatively impact posttransplant survival [19–21]. Hayes et al. [34] assessed the impact of PH on survival in patients with IPF following LT. They compared survival rates between patients with mPAP < 25 mmHg versus ≥ 25 mmHg over 84 months, reporting median survival times of 60.4 months (95% CI: 55.2–80.4) and 61.4 months (95% CI: 56.9–66.9), respectively (*p*=0.876). Similar to our findings, their study confirmed that PH is prevalent among these patients, but there is no substantial evidence to suggest that PH significantly alters the risk of death after LT in PF patients.

Wu et al. [35] analyzed survival in patients undergoing LT due to end-stage lung disease with PH, finding a 1-year survival rate of 80%, a 3-year survival rate of 65%, and a 5-year survival rate of 50%. The presence of severe PH did not emerge as a determinant factor for poorer survival outcomes in LT recipients [35]. In contrast, our study showed a higher survival rate at both 1 year and 5 years of follow-up, without a significant decline in survival regardless of the presence of PH.

Thomas et al. [36] reported a PH prevalence of 27.9% among 245 patients listed for LT due to end-stage lung disease related to SARS-CoV-2 infection, with a prevalence of 31.0% in those who ultimately underwent transplantation. Survival for patients without PH was 96.4% (95% CI: 89.0%–98.8%) at 120 days, compared to 95.7% (95% CI: 84.0%–98.9%) for those with PH (*p*=0.953). Our cohort did not include patients with COVID-19-related PF, but when comparing the two cohorts, no statistical differences in survival, either before or after LT, were observed based on the presence of PH.

Patients with PH may exhibit a reduced distance covered during the 6MWT due to increased pulmonary vascular resistance and impaired functional capacity [37–39]. Furthermore, quality of life is often diminished as a result of the decreased ability to perform daily activities and engage in physical exercise [40, 41]. Our results showed a lower average distance covered in the 6MWT and higher SGRQ total scores in patients diagnosed with PH.

In our study, we observed a progressive improvement in FVC, FEV1, and 6MWT performance in the PH group, from pre-LT to hospital discharge, and continuing through 3, 6, and 12 months posttransplant. This phenomenon can be attributed to the removal of diseased tissue, replacement with healthy tissue, reduction in pulmonary vascular pressures, postoperative rehabilitation, and management of comorbidities, all of which contribute to improved pulmonary function and physical capacity [42–46].

Singer et al. reported a significant average decrease of 47 points in the SGRQ score among LT recipients over 5 years posttransplant [47]. Furukawa et al. found that in nontransplanted PF patients, an SGRQ score above 30 was an independent prognostic factor associated with higher mortality [48]. Regarding quality of life, our findings showed a progressive reduction in SGRQ total scores from pre-LT to 5 years posttransplant, from 58.06 points to 16.48 points.

Kim et al. [49] examined the impact of PH diagnosed prior to LT on survival, finding a 1-year survival rate of 58.8% in patients with mPAP ≥ 25 mmHg compared to 87.5% in those with mPAP < 25 mmHg. They also noted that PH was associated with increased postoperative complications. Similarly, Fang et al. [50] demonstrated that elevated mPAP was associated with primary graft dysfunction. Consistent with these studies, our findings also showed a higher incidence of surgical and early postoperative complications in patients with PH.

The results of our study provide valuable insight into the outcomes of LT in patients with PH at a high-altitude location, where oxygen levels are lower, presenting a unique physiological challenge, especially for patients with pulmonary conditions such as PH [51–53]. The body's adaptation to hypoxia at high altitudes generally involves an increase in red blood cell production and alterations in pulmonary and cardiovascular responses, which could influence posttransplant recovery [51–54]. The effects of living at high altitudes on LT outcomes remain underexplored, making this study particularly valuable [51, 53, 55]. Our findings highlight the importance of considering altitude-related factors when evaluating transplant outcomes in such settings. Given the limited research on this topic, especially in Latin America, our study provides novel data that could inform clinical practices and improve the management of lung transplant recipients in high-altitude regions, a perspective that is underrepresented in current medical literature.

### 4.1. Limitations

The strengths of this study lie in its prospective design, rigorous methodology, and data collection by intensive care staff trained in LT. It is important to note that mPAP measurement was performed in all cases through cardiac catheterization. Among the study's limitations are the small sample size and the inclusion of patients from a single hospital. The severity of PF was not assessed, which may have introduced bias when evaluating the MCID in lung function, as measured by FEV1 and FVC, between patients with and without PH. This is because the stiffness and reduced elasticity of fibrotic lung tissue can significantly impact lung function [56, 57]. Additionally, the PH group had a higher preoperative comorbidity burden compared to the control group, which may limit the generalizability of our findings. Another limitation is the lack of a low-altitude comparison group, which limits the extrapolation of our results to other populations living at sea level or at lower elevations. Therefore, more studies are needed to compare lung transplant patients with PF at different altitudes to better understand how altitude affects transplant outcomes.

## 5. Conclusion

Survival at 1 and 5 years was lower in patients with PH. No significant differences in survival were found based on sex, age, or BMI. On the other hand, statistically significant improvements were observed in FVC, FEV1, 6MWT, and SGRQ measurements both before and after LT, which continued through 1 year of clinical follow-up. The 6MWT and SGRQ total score showed MCID before the surgical procedure and during clinical follow-up at 6 and 12 months among patients with and without PH. The frequency of intraoperative and immediate complications was higher in the PH group.

## Figures and Tables

**Table 1:** General characteristics of the population.

	PH *n* = 32	No PH *n* = 7
Age, mean (SD)	52.6 (10.2)	48.6 (10.6)
Male, *n* (%)	16 (50)	4 (57)
BMI, mean (SD)	25.6 (3.9)	24.8 (2.5)
mPAP, mean (SD)	31.1 (6.4)	17.6 (1.4)

*Diagnosis of pulmonary fibrosis, n (%)*		
Allergic alveolitis	2 (6)	0 (0)
Sjogren's syndrome	0 (0)	2 (29)
Rheumatoid lung	2 (6)	0 (0)
Sarcoidosis	1 (3)	0 (0)
Hypersensitivity pneumonitis	4 (12)	0 (0)
NSIP	11 (34)	1 (14)
UIP	7 (22)	3 (43)
ARDS	0 (0)	1 (14)
Lymphangiomyomatosis	5 (16)	0 (0)

*Note:* noninfectious nonspecific interstitial pneumonia (NSIP), Pulmonary Fibrosis of Indeterminate Origin (UIP).

Abbreviations: ARDS, acute respiratory distress syndrome; BMI, body mass index; mPAP, mean pulmonary arterial pressure; PH, pulmonary hypertension; SD, standard deviation.

**Table 2:** Comparison of transplantation outcomes between pulmonary and no pulmonary hypertension.

	PH *n* = 32	No PH *n* = 7
*Transplant, n (%)*		
Unipulmonary	5 (16)	0 (0)
Two-lung	27 (84)	7 (100)

Ischemia LL minutes, m (IQR)	278 (240–300)	306 (250–405)
Ischemia RL minutes, m (IQR)	360 (300–393)	390 (360–470)
Surgical time minutes, m (IQR)	340 (305–385)	354 (330–420)

CCE, *n* (%)	3 (9)	0 (0)
ECMO, *n* (%)	8 (25)	1 (14)

*Intrasurgical complications, n (%)*		
None	21 (67)	7 (100)
mPAP > 20 mmHg	4 (12)	0 (0)
Bleeding	3 (9)	0 (0)
Arrhythmias	2 (6)	0 (0)
Tear or lost anastomosis	1 (3)	0 (0)
Right ventricular dysfunction	1 (3)	0 (0)

Blood products, *n* (%)	21 (66)	5 (71)
Inotropes, *n* (%)	32 (100)	7 (100)
IMV days, m (IQR)	4 (3–8)	6 (4–7)
Days of stay CCU, m (IQR)	12 (7.5–18)	11 (9–30)

*Immediate complications, n (%)*		
None	21 (67)	6 (86)
Primary graft dysfunction	3 (9)	0 (0)
mPAP > 20 mmHg	2 (6)	1 (14)
Bleeding	2 (6)	0 (0)
Right ventricular dysfunction	1 (3)	0 (0)
Pancreatitis	1 (3)	0 (0)
Aortic infection	1 (3)	0 (0)
Hemothorax	1 (3)	0 (0)

*Late complications, n (%)*		
None	16 (50)	2 (29)
Aspergillosis	5 (15)	0 (0)
Tuberculosis	4 (13)	0 (0)
Bronchial stenosis	1 (3)	0 (0)
Pseudomonas aeruginosa	0 (0)	2 (29)
Community-acquired pneumonia	2 (6)	2 (29)
Chronic rejection	4 (13)	1 (13)

Abbreviations: CCU, critical care unit; ECC, extracorporeal circulation; ECMO, extracorporeal membrane oxygenation; IMV, invasive mechanical ventilation; IQR, interquartile range; LL, left lung; m, median; mPAP, mean pulmonary arterial pressure; PH, pulmonary hypertension; RL, right lung.

**Table 3:** Functional tests and quality of life.

	Functional variables	Pretransplant	Hospital discharge posttransplant	3 months posttransplant	6 months posttransplant	12 months posttransplant	*p* value
PH	FVC L	2.09 (1.5–2.7)	2.18 (1.9–2.8)	2.7 (2.1–3.3)	2.98 (2.5–3.5)	2.92 (2.3–3.4)	< 0.001
No PH	m (IQR)	1.92 (1.4–2.8)	1.77 (1.6–2.6)	2.27 (2–2.7)	2.7 (2.5–3.2)	2.88 (2.5–3.7)	0.001

PH	FEV_1_ L	1.41 (1.1–2.1)	1.76 (1.4–2.3)	2.37 (1.7–2.8)	2.47 (2.1–3)	2.42 (1.6–2.8)	< 0.001
No PH	m (IQR)	1.35 (1–1.6)	1.54 (1.3–2.3)	1.87 (1.6–2.5)	2.4 (1.9–2.8)	2.71 (1.9–3.5)	0.005

PH	6MWT	480 (401–555)	296.5 (195–395)	521.5 (471.5–562.5)	559.5 (505–610)	567.5 (442–611)	< 0.001
No PH	m (IQR)	535 (420–586)	373 (271–379)	511 (439–587)	584 (424–633)	607 (595–637)	< 0.001

PH	SGRQ total	58.06 (44.5–68.5)	26.11 (11.6–34.3)	19.66 (8.1–33.5)	14.06 (5.5–25.5)	16.48 (6.5–31.6)	< 0.001
No PH	m (IQR)	53.18 (48.1–68)	43.65 (25–57.4)	15.06 (10.8–28.3)	8.5 (4.9–15.4)	4.24 (2.5–12.6)	0.001

*Note:* forced expiratory volume in the first second (FEV1).

Abbreviations: 6MWT, Six-Minute Walk Test; FVC, forced vital capacity; IQR, interquartile range; m, median; SGRQ, St George's Respiratory Questionnaire.

## Data Availability

The data that support the findings of this study are available from the corresponding author upon reasonable request.

## Supporting Information

Supporting Information 1 In Supporting Table 1, the post hoc analysis of the Pulmonary Function Tests, Six-Minute Walk Test, and Quality of Life is described. Subsequently, a post hoc analysis was conducted using the Wilcoxon test or the Nemenyi test.

Supporting Information 2 In Supporting Table 2, the minimal clinically important differences (MCID) for Pulmonary Function Tests, Six-Minute Walk Test (6MWT), and Quality of Life are described. The minimal clinically important difference (MCID) for functional variables was established before transplantation, at hospital discharge and during clinical follow-up for patients without and with PH: an increase in forced expiratory volume in the first second greater than 100 mL, in FVC greater than 200 mL or 10%, a change in the distance covered in the 6MWT test greater than 30 m, and an improvement in quality of life according to the St. George's Respiratory Questionnaire greater than 4 units.
