# Supplementary material for: Impact of Pulmonary Hypertension on Posttransplant Survival of Patients With Pulmonary Fibrosis at High Altitude: A Prospective Cohort Study
Source: Can Respir J. 2025 Feb 24;2025:1861990. doi: 10.1155/carj/1861990 (PMC11876537; doi:10.1155/carj/1861990)
Supplement: Supporting Information 1 — In Supporting Table 1, the post hoc analysis of the Pulmonary Function Tests, Six-Minute Walk Test, and Quality of Life is described. Subsequently, a post hoc analysis was conducted using the Wilcoxon test or the Nemenyi test. [file 1861990.f1.pdf]

Supplementary table 1. Post hoc Analysis of Pulmonary Function Tests, Six-Minute Walk Distance, and Quality of Life.

	FVC		FEV_1		6MWT		SGQR total	
	PH n=32	No PH n=7	PH n=32	No PH n=7	PH n=32	No PH n=7	PH n=32	No PH n=7
Pre-transplant / 3 months*	0.004	0.176	<0.001	0.236	0.572	0.735	<0.001	0.018
Pre-transplant / 6 months*	<0.001	0.028	<0.001	0.091	0.009	0.028	<0.001	0.018
Pre-transplant / 12 months*	0.002	0.018	<0.001	0.043	0.144	0.018	<0.001	0.018
Pre-transplant / Hospital discharge*	0.065	0.866	0.004	0.128	<0.001	0.018	<0.001	0.091
3 months* / 6 months*	0.115	0.018	0.043	0.018	0.001	0.075	0.469	0.128
3 months* / 12 months*	0.328	0.018	0.485	0.018	0.057	0.018	0.809	0.018
3 months* / Hospital discharge*	0.003	0.499	0.003	0.398	<0.001	0.028	0.032	0.063
6 months* / 12 months*	0.925	0.063	0.741	0.091	0.904	0.063	0.469	0.735
6 months* / Hospital discharge*	0.001	0.043	0.002	0.176	<0.001	0.018	0.014	0.043
12 months* / Hospital discharge*	0.013	0.018	0.031	0.091	<0.001	0.018	0.17	0.028

Notes: Pulmonary hypertension (PH), Forced Vital Capacity (FVC), Forced Expiratory Volume in the First Second (FEV1), Six-Minute Walk Test (6MWT), St George's Respiratory Questionnaire (SGRQ); *: post-transplant.
