# Supplementary material for: Impact of Pulmonary Hypertension on Posttransplant Survival of Patients With Pulmonary Fibrosis at High Altitude: A Prospective Cohort Study
Source: Can Respir J. 2025 Feb 24;2025:1861990. doi: 10.1155/carj/1861990 (PMC11876537; doi:10.1155/carj/1861990)
Supplement: Supporting Information 2 — In Supporting Table 2, the minimal clinically important differences (MCID) for Pulmonary Function Tests, Six-Minute Walk Test (6MWT), and Quality of Life are described. The minimal clinically important difference (MCID) for functional variables was established before transplantation, at hospital discharge and during clinical follow-up for patients without and with PH: an increase in forced expiratory volume in the first second greater than 100 mL, in FVC greater than 200 mL or 10%, a change in the distance covered in the 6MWT test greater than 30 m, and an improvement in quality of life according to the St. George's Respiratory Questionnaire greater than 4 units. [file 1861990.f2.pdf]

Supplementary table 2. minimal clinically important difference for functional variables.

	FVC L, mean	FEV_1 L, mean	6MWT m, mean	SGRQ total, mean
Pre-trasplant	-0,170	-0,060	55	-4,8
Hospital discharge post-transplant	-0,405	-0,220	76,5	17,5
3 months post-transplant	-0,430	-0,500	-10,5	-4,6
6 months post-transplant	-0,280	-0,065	24,5	-5,5
12 months post-transplant	-0,040	0,295	39,5	-12,2

Notes: Forced Vital Capacity (FVC), Forced Expiratory Volume in the First Second (FEV1), Six-Minute Walk Test (6MWD), St George's Respiratory Questionnaire (SGRQ).
